# Elevated Autoantibodies to the GluA1 Subunit of the AMPA Receptor in Blood Indicate Risk of Cognitive Impairment in Contact Sports Athletes, Irrespective of Concussion

**DOI:** 10.1089/neur.2023.0132

**Published:** 2024-06-06

**Authors:** Christopher Bailey, Daniel Soden, Joseph Maroon, Warren Selman, Christopher Tangen, John Gunstad, Susannah Briskin, Shana Miskovsky, Emiko Miller, Andrew A. Pieper

**Affiliations:** ^1^ Department of Neurology, School of Medicine, Case Western Reserve University, Cleveland, Ohio, USA.; ^2^ University Hospitals Sports Medicine Concussion Center, University Hospital Cleveland Medical Center, Cleveland, Ohio, USA.; ^3^ Department of Neurological Surgery, University of Pittsburgh Medical Center, Pittsburgh, Pennsylvania, USA.; ^4^ Department of Psychological Sciences, Kent State University, Kent, Ohio, USA.; ^5^ Brain Health Medicines Center, Harrington Discovery Institute, University Hospitals Cleveland Medical Center, Cleveland, Ohio, USA.; ^6^ Department of Psychiatry, Case Western Reserve University School of Medicine, Cleveland, Ohio, USA.; ^7^ Geriatric Psychiatry, GRECC, Louis Stokes VA Medical Center, Cleveland, Ohio, USA.; ^8^ Institute for Transformative Molecular Medicine, School of Medicine, Case Western Reserve University School of Medicine, Cleveland, Ohio, USA.; ^9^ Department of Neurosciences, Case Western Reserve University School of Medicine, Cleveland, Ohio, USA.; ^10^ Department of Pathology, Case Western Reserve University School of Medicine, Cleveland, Ohio, USA.

**Keywords:** AMPAR, cognition, concussion, contact sports, fluid biomarker, GluA1, reaction time

## Abstract

To address the need for objective tests of concussion in athletes, we conducted a prospective clinical study in National Collegiate Athletic Association athletes of the relationship between neurocognitive performance and blood levels of the GluA1 subunit of α-amino-3-hydroxy-5-methyl-4-isoxazolepropionic acid receptor peptides and autoantibodies to GluA1. Specifically, we compared 44 contact sport athletes to 16 noncontact sport athletes, with Immediate Post-Concussion Assessment and Cognitive Testing (ImPACT), as well as blood sample collection, before the start of the season and at the end of the season. Contact sport athletes exhibited significantly elevated serum GluA1 autoantibodies at the end of season, compared with preseason levels, irrespective of whether they sustained a concussion. Noncontact sport athletes showed no change in serum GluA1 autoantibodies, and neither group showed differences in GluA1 peptides. Amongst contact-sport athletes, the ‘high GluA1 autoantibody group’ (≥4 ng/mL) displayed impaired reaction time, a measure of cognitive impairment, while the ‘low GluA1 autoantibody group’ (<4 ng/mL) displayed normal reaction time. Our results reveal that contact sport athletes are at risk for developing cognitive impairment even without sustaining a diagnosed concussion and that serum GluA1 autoantibodies provide a blood-based biomarker of this risk. This could guide future studies on the differing susceptibility to cognitive impairment in contact sport athletes and facilitate efficient allocation of resources to contact sport athletes identified as having increased risk of developing cognitive impairment.

## Introduction

The evaluation and diagnosis of concussion, a highly prevalent form of traumatic brain injury (TBI), has become an increasingly well-recognized public health concern in recent years.^1,2^ Indeed, at present 1.2% of people are diagnosed with a concussion every year.^3^ In addition to acute complications, TBI causes progressive and chronic axonal degeneration^4–8^ and increased risk of developing aging-related neurodegenerative diseases, including Alzheimer’s disease and Parkinson’s disease.^9^ TBI is a particularly prominent problem in contact sports, and National Collegiate Athletic Association (NCAA) athletes sustain more than 10,000 concussions per year, representing 6.2% of injuries among NCAA athletes.^10^ The frequency of concussion has galvanized multiple consensus statements, informing initial diagnosis and subsequent treatment.^11,12^ Because most concussions do not result in visible injury or abnormalities on clinically available neuroimaging (e.g., CT scan or MRI), the diagnosis and management of concussion rely primarily on subjective symptom report.^12,13^ Unfortunately, the heterogeneous and nonspecific nature of concussion symptoms greatly complicates diagnosis and treatment of patients. In addition, external factors (e.g., desire to return to sport or secondary gain) can lead to inaccurate reporting of symptoms, further complicating diagnosis and treatment.^14^

While impairment in cognitive performance can be caused by concussion, it is not known whether concussion is absolutely required for the development of cognitive impairment in people subjected to chronic and repeated lower levels of head impact, such as can occur in contact sports. Coupled with the under-reporting and underdiagnosis of concussion, this creates an unmet need for identifying people in high-contact situations who are at greatest risk of developing cognitive impairment, irrespective of their officially reported medical history, to direct the appropriate resources for long-term brain health.^15^ A multidisciplinary approach (e.g., neurocognitive, vestibular, and oculomotor testing) is often utilized in attempts to identify these individuals, but these procedures are resource intensive and often subjectively interpreted. Biomarkers, on the other hand, provide objective data that improve accuracy and enable optimized delivery of appropriate treatments.

As an actively developing field, fluid biomarkers have demonstrated utility in identifying head injury.^16,17^ For example, multiple blood biomarkers have been investigated in TBI, such as S-100β, glial fibrillary acidic protein (GFAP), neuron-specific enolase, neurofilament light (NfL), and ubiquitin C-terminal hydrolase L1.^18–21^ However, their clinical utility has been limited to date.^21–23^ Recently, acetylated-tau was also identified as a serum biomarker for acute and chronic neurodegeneration after TBI, which uniquely represents the abundance of a therapeutic target in the brain for mitigating neurodegeneration.^24^ However, there is still additional need for biomarkers that may identify risk for cognitive decline, irrespective of the formal diagnosis of any form of TBI.^25^

Within a sports context, previous studies have suggested a cumulative effect of subconcussive impacts on cognition. For example, a recent systematic review by Walter and colleagues identified increases in fluid biomarkers of brain injury over the course of a season for athletes in contact sports.^26^ While cognitive functioning did not typically change over the course of a season, methodological differences made it difficult to draw conclusions about the outcome. In addition, Kawata and colleagues discovered increased plasma S100β in collegiate football players over the course of a season, even though players did not endorse symptoms of concussion.^27^ In a separate study examining elevations in microRNA as related to cognitive functioning, Papa and colleagues discovered elevated amounts of microRNAs in collegiate football players, compared with control subjects. They also demonstrated increased levels of microRNAs postseason for the football players, with select microRNAs associated with impaired reaction time and balance.^28^ More recently, blood-based biomarkers (GFAP, total Tau, and NfL) were also noted to increase over the course of a single football season, irrespective of concussion diagnosis, with highest increases noted in player positions associated with low-frequency high-magnitude impacts.^29^

The presence of serum peptides and autoantibodies to the ionotropic type glutamate receptor (α-amino-3-hydroxy-5-methyl-4-isoxazolepropionic acid receptor [AMPAR]) has recently been proposed as a biomarker of TBI, in studies that included analysis of AMPAR serum peptides and autoantibodies involving groups consisting of GluA1/GluA6_7, GluA2/GluA6_7, GluA3/GluA6_7, GluA1/GluA6, GluA2/GluA7, and any combination thereof in the biological sample.^30–32^ AMPARs are primarily distributed in the forebrain and subcortical pathways, which are highly vulnerable to mild brain injury.^33^ Indeed, there is a massive release of glutamate during the acute phase of mild TBI, which upregulates the expression of excitotoxic AMPARs. In addition, after brain injury the N-terminal of the GluA1 subunits of AMPARs is cleaved by extracellular proteases and released into the bloodstream through the compromised blood–brain barrier.^33^ This GluA1 peptide was recently shown to be acutely and significantly elevated after concussion in club sport athletes,^30^ and serum GluA1 peptide values correlate with acute computerized concussion testing (Immediate Post-Concussion Assessment and Cognitive Testing [ImPACT]) after concussion.^30^

In this study, we have investigated whether blood levels of GluA1 autoantibodies are more highly elevated at the end of the season in contact sport athletes compared with noncontact sport athletes, irrespective of diagnosis of concussion. If this proved to be the case, then our study was additionally designed to determine whether the level of GluA1 autoantibody could be used to predict risk of cognitive impairment. Specifically, we examined pre- and postseason levels of GluA1 peptides and autoantibodies in a college athlete population, with respect to measures of cognitive impairment. As shown here, we confirmed that contact sport athletes are at risk for developing cognitive impairment even without ever sustaining a concussion and that serum GluA1 autoantibodies provide a blood-based biomarker of this risk.

## Materials and Methods

### Study participants

**FIG. 1.**
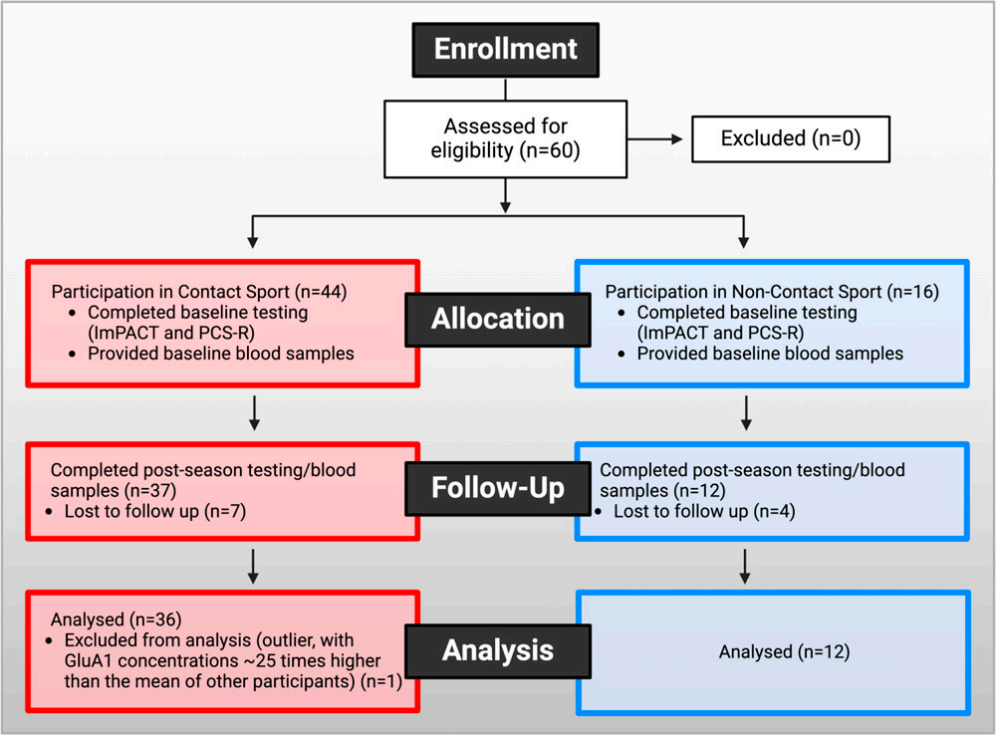
CONSORT diagram of study subject enrollment in the study. All 60 participants were eligible, and thus, none was excluded. Forty-four of these participants were in contact sport, and 16 were in noncontact sport. All participants in both groups completed baseline ImPACT and PCS-R testing and provided baseline blood samples. In the contact sport group, 7 participants were lost to follow-up and 37 completed postseason baseline ImPACT and PCS-R testing and provided blood samples. In the noncontact sport group, 4 participants were lost to follow-up and 12 completed postseason baseline ImPACT and PCS-R testing and provided blood samples. In the contact sport group, one subject was excluded from analysis because they were an outlier with GluA1 concentrations ∼25 times greater than the mean of other participants. No subjects were excluded from the noncontact sport group. ImPACT, Immediate Post-Concussion Assessment and Cognitive; PCS-R, Post-Concussive Scale-Revised.

**FIG. 2.**
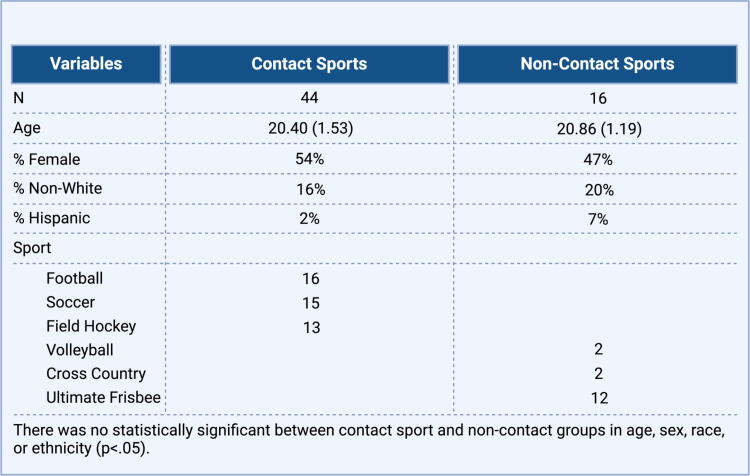
Demographic characteristics of subjects participating in the study. Contact sport participants included 16 in football, 15 in soccer, and 13 in field hockey. Noncontact sport participants included 2 in volleyball, 2 in cross-country, and 12 in ultimate frisbee. There were no statistically significant differences between contact and noncontact sport groups in age, sex, race, or ethnicity.

Participants were recruited from team meetings and other team events prior to the beginning of contact practice at two local universities (1 NCAA Division I and 1 NCAA Division III). Inclusion criteria were participation in a varsity college sport, being at least 18 years of age, and participation in a contact sport (football, soccer, or field hockey) or a noncontact sport (volleyball, cross-country, or ultimate frisbee). Participants were excluded from the study if venipuncture was not feasible, if they had a diagnosis of ischemic or hemorrhagic stroke or other neurological disorder outside of concussion/mild TBI (e.g., moderate-severe TBI, dementia, Parkinson’s disease, multiple sclerosis, seizure disorder, brain tumors), or if they had a history of neurosurgery within the last year. Sixty athletes met criteria for study inclusion. One participant was excluded from the final sample as an outlier, with GluA1 concentrations that were approximately 25 times higher than the mean across groups. An additional 11 participants were lost to follow-up. The final sample contained 48 participants, with 36 in the contact sport group and 12 in the noncontact sport group. The full CONSORT diagram is provided in Figure 1, and participant characteristics are provided in Figure 2. The study was approved by the academic medical IRB, and informed consent was obtained from all participants prior to data collection.

### Procedures

All student athletes underwent preseason physicals and standard clinical procedures for baseline concussion testing prior to the start of practice, which included ImPACT^34,35^ and Post-Concussive Scale-Revised (PCS-R).^36^ All participants also completed questionnaires for assessment of symptoms (including mental health and injury related questions) and had blood draws (up to 8 mL). ImPACT, PCS-R, and blood draw procedures were also completed at the conclusion of the athletic season. If athletes sustained a concussion during the season, standard concussion evaluation methods were used (including acute postinjury SCAT5 testing and daily symptom scale completion/symptom monitoring), and blood samples were collected within 24–48 hours of the injury. Return to sport was based on physician clearance according to concussion symptom resolution (at rest and with exercise), as well as return to preseason levels of performance on SCAT5 and ImPACT. At the time of the clearance evaluation for the concussed athlete, participants repeated a combination of computerized concussion testing (ImPACT) and traditional neurocognitive testing, and a final sample of blood was taken. Thirty-seven percent of the total sample (22 of 60 participants) reported a history of previously diagnosed concussions, with 25% (4 of 16) of the noncontact sport group and 41% (18 of 44) of the contact sport group reporting a history of concussion. During the study, only three cases (identified in Fig. 7) were diagnosed by their sports medicine staff as having sustained a concussion. The rest of the subjects were not diagnosed with having sustained a concussion during the sports season. The three concussed subjects were analyzed separately and excluded from the analyses with the contact and noncontact groups.

### Blood sample collection, storage, and shipping

Plasma in EDTA containing tubes (usually closed with purple cap) was collected, while serum was drawn into tubes filled with gel used for sample clotting (yellow cap). After centrifugation of the tubes at 3,500 g for 5 min at 4°C, plasma (clear liquid on the top) from the purple cap tube was separated from red blood cells that had concentrated at the bottom of the tube. Serum samples were collected by pipette from the top of serum tube and transferred into Eppendorf tubes, which were marked with an alphanumeric indicator. Samples were stored at −20°C for up to 3 weeks, and at –70°C thereafter until assayed. Once ready to be received and processed, samples were shipped in a transport box (Fisher Scientific Catalog #5954 −5″ × 5″ box) that could hold up to 80 samples and dry ice. Blinded samples were shipped to Grace Labs, LLC for GluA1 processing. Plasma samples are a good choice for peptide and protein biomarker detection and are better drawn in antipeptidase-treated tubes to protect labile peptides. Various peptide biomarkers have different sensitivities to coagulants (heparin, EDTA, and citric acid). Compared to plasma, serum samples contain about 20–30% more antibodies and are therefore useful for antibody biomarker measurements.^37^

### Outcome measures

Primary measures included fluid biomarker concentrations (i.e., GluA1 autoantibodies and peptides), neurocognitive testing (ImPACT), and symptom endorsement (as measured by the PCS-R). GluA1 peptides and antibodies were examined at baseline and upon completion of the season. Cognitive testing was examined in conjunction with biomarker concentrations to determine if GluA1 autoantibody and peptide elevations were associated with impaired neurocognitive functioning.

### GluA1 peptide assay

Briefly, 20 μL plasma samples, five calibrators, negative/positive controls in duplicates, and 80 μL of working mixture consisting of magnetic particles (MP) with covalently attached specific antibodies against GluA1 peptide were added to the microtiter plate. The mixture was incubated for 2 min at 37°C; GluA1 antibody-labeled horseradish peroxidase (HRP) solution was then added for 20 min at 37°C. After the bound magnetic particles were washed with a buffer using a magnetic separator, the reaction was revealed by pipetting 100 μL ready-to-use tetramethyl benzidine (TMB) substrate into each well of the microtiter plate. The color reaction was developed for 8 min at 25°C, stopped with acid solution (100 μL), and monitored at 490/630 nm on a microplate reader (Bio Tek ELx800™, BioTek Instruments, Inc.). GluA1 peptide concentrations in plasma were determined by plotting their absorbance values on a calibration curve constructed from the absorbance units of each calibrator and their known concentrations. The intra-assay coefficient variation (CV) was 5.1–6.2%, and the interassay CV was 5.7–9.5%.

### GluA1 Ab assay

We added 100 μL of diluted blood sera (1:50) and sets of calibrators to GluA1 peptide-coated wells of microplates and incubated the plates for 30 min at 37°C. After the wells were washed with buffer, 100 μL of rabbit antihuman horseradish peroxidase-labeled IgG (1:1000 dilution) was added to the wells and incubated for 30 min. After additional washing, detection solution (*o*-phenylenediamine in 0.05 mol/L citrate buffer, pH 4.3) was pipetted (100 μL) into each well of the microplate. The color reaction was developed for 10 min, stopped with acid solution (30 μL), and monitored at 490 nm on a microplate reader. Sample buffer was also included as the blank in each assay to calculate the zero-unit value. GluA1 Ab in serum was determined by use of the calibration curve of absorbance units of Abs versus concentration in the microplate wells.

### Statistics and data analysis

Interval change in all analyses was calculated by subtracting the baseline value from the postseason value for each analysis, meaning that a positive value represents an increase relative to baseline and a negative value represents a decrease relative to baseline.

## Results

**FIG. 3.**
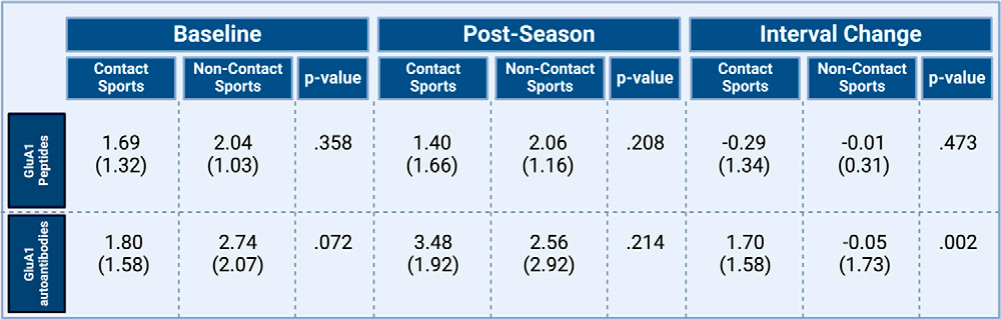
Concentration of serum GluA1 autoantibodies and plasma GluA1 peptides (ng/mL) at baseline and postseason and determination of interval change. There were no significant changes across groups.

**FIG. 4.**
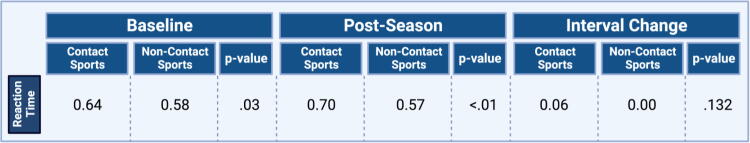
ImPACT reaction time of study subjects pre- and postseason. Reaction time worsened postseason in contact sport athletes, with the mean change in reaction time approaching statistical significance. ImPACT, Immediate Post-Concussion Assessment and Cognitive.

**FIG. 5.**
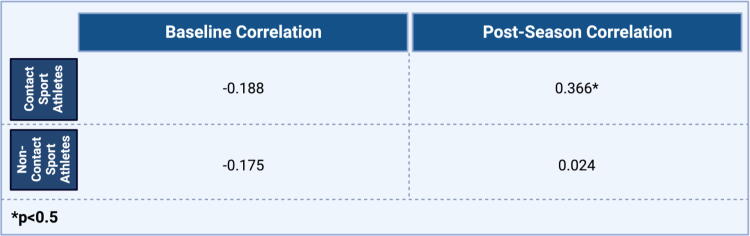
Correlation of reaction time and serum GluA1 autoantibodies pre- and postseason. A bivariate Pearson correlation was completed **(r)**. The ImPACT reaction time was poorly correlated with serum GluA1 autoantibodies. Slowed reaction time related to increase GluA1 autoantibodies only in the contact sport group at the end of season. ImPACT, Immediate Post-Concussion Assessment and Cognitive.

Over the course of the season, contact sport athletes demonstrated greater increases in serum GluA1 antibodies compared with noncontact sport athletes [*t*(46)=3.24; *p* = 0.002]. However, no significant changes across groups were noted in plasma GluA1 peptides (Fig. 3). Overall, reaction time worsened postseason in contact sport athletes, with the mean change in reaction time (contact: M = 0.06, SD = 0.19; low contact: M = 0.00, SD = 0.04) approaching significance [t(37.23)=1.54; *p* = 0.132; Figure 4]. In addition, ImPACT reaction time was poorly correlated (r = .37; *p* = 0.043) with serum GluA1 autoantibodies, with slowed reaction time related to increased GluA1 autoantibodies only in contact athletes at the end of the season (Fig. 5).

**FIG. 6.**
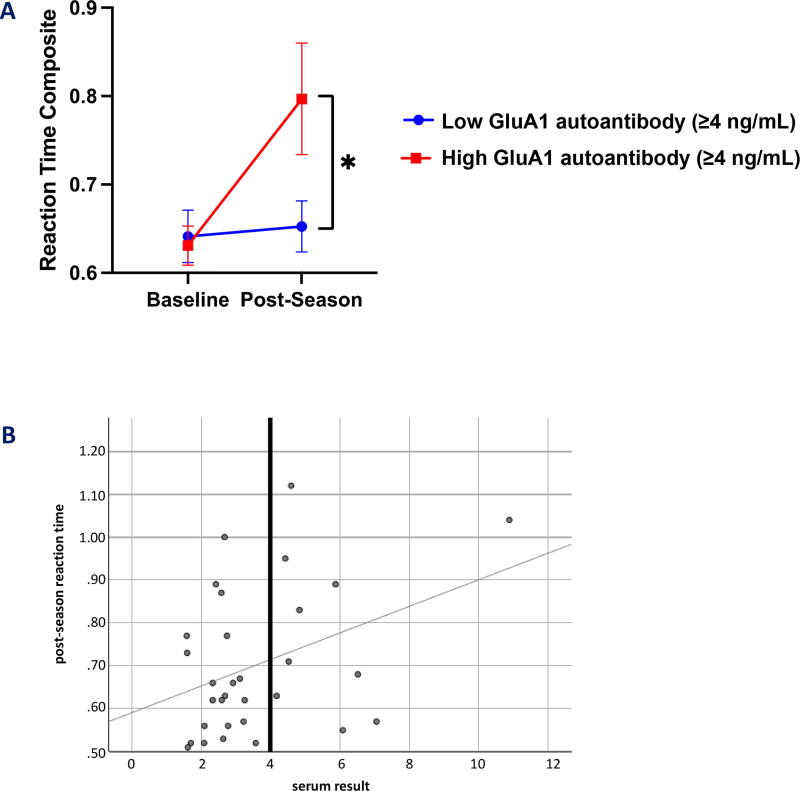
**(A)** Reaction time in high versus low GluA1 autoantibody groups in contact sport athletes shows that high GluA1 autoantibody is associated with cognitive impairment. **(B)** Scatter plot of postseason reaction time (*y*-axis) versus serum result (ng/mL GluA1 autoantibody) illustrates how 4 ng/mL was selected based on the visualized separation in the data between those above and below the 4 ng/mL threshold.

**FIG. 7.**
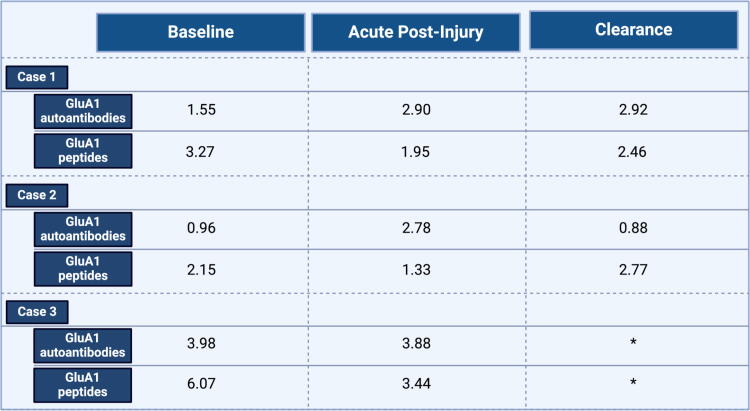
Concentration of serum GluA1 autoantibodies and plasma GluA1 peptides in the three participants who sustained a concussion, at baseline, acutely postinjury, and time of clearance (if cleared). The * indicates that concussion case 3 was not cleared, as they left the area and withdrew from the study. These three concussed subjects were analyzed separately and excluded from the analyses with the contact and noncontact groups.

Given the elevations in GluA1 autoantibodies and strong trend toward impaired reaction time in contact sport athletes at the end of season, we further separated contact athletes into low (<4 ng/mL) and high (≥4 ng/mL) GluA1 autoantibody groups at the postseason time point (Fig. 6). Athletes in the high GluA1 autoantibody group demonstrated significantly higher degrees of change in reaction time compared with those in the low GluA1 autoantibody group (*p* < 0.05, repeated measures ANOVA interaction group (high vs. low AMPAR group) × time point; f(30)=5.01; *p* = 0.033; partial η2 = 0.14). Contact sport athletes in the high GluA1 autoantibody group also demonstrated clinically meaningful worsening in ImPACT reaction time composite (mean change in reaction time = 0.17; far exceeding the 99% confidence interval for reliable decline), whereas contact sport athletes in the low GluA1 autoantibody group showed no change (Fig. 6). In the three cases of concussion, serum GluA1 autoantibody levels increased as expected, while increased plasma GluA1 peptides were not observed (Fig. 7). These cases were excluded from analysis of contact versus noncontact sports groups.

## Discussion

**FIG. 8.**
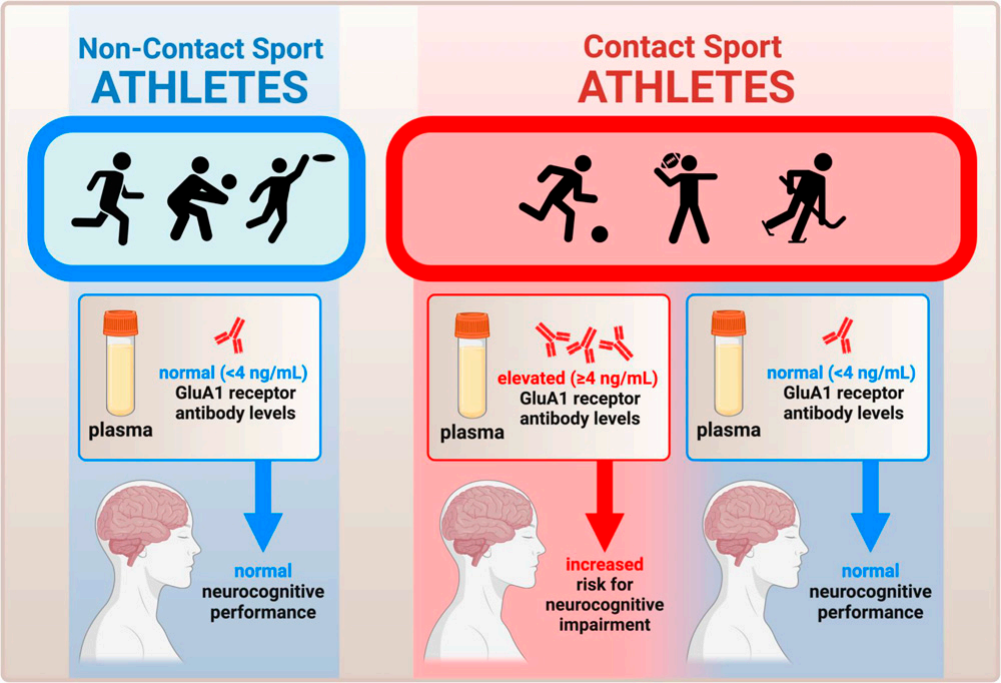
Summary schematic of the study, illustrating the major finding that GluA1 antibodies are selectively elevated in contact sport athletes relative to noncontact sport athletes, irrespective of concussion, and that within contact sport athletes the magnitude of serum GluA1 autoantibodies indicates the risk of developing cognitive impairment.

This study examined GluA1 autoantibodies and peptides in both contact sport and noncontact sport athletes, and few formally diagnosed concussions occurred over the course of the season. In the three cases of concussion, serum GluA1 autoantibody levels increased as expected, while increased plasma GluA1 peptides were not observed. However, GluA1 autoantibody levels also increased at the end of the season in contact sport athletes, compared with noncontact sport athletes, irrespective of whether they sustained a concussion. No significant findings were observed in GluA1 plasma peptides across groups. In addition, GluA1 autoantibody elevations correlated to neurocognitive testing (ImPACT reaction time) at the postseason time point and in contact sport athletes. Specifically, contact-sport athletes with a high level of GluA1 autoantibodies (≥4 ng/mL) showed impaired reaction time, which was not observed in contact sport athletes in the low GluA1 autoantibody range (<4 ng/mL) (Fig. 8). The average change in reaction time for the high GluA1 autoantibody group was clinically significant (approximately thrice the amount needed for identification as a clinically reliable decline on ImPACT when utilizing an 80% confidence interval).^35^ Importantly, no concussions were reported in the high GluA1 autoantibody group, despite impaired reaction time findings, confirming that chronic head injury with cognitive impairment can be caused in college athletes over the course of a season without sustaining a concussion. Finally, GluA1 autoantibodies provide a clinically meaningful blood biomarker of impaired cognition in contact sport athletes in our sample, independent of concussion.

Although we observed elevated GluA1 autoantibodies in contact sport athletes, we did not detect elevated GluA1 peptides, as might have been expected from the literature.^28–30^ Although collection and storage procedures were followed appropriately, we cannot rule out the possibility of degradation of GluA1 peptides prior to processing. Alternatively, elevated autoantibodies to GluA1 without changes in GluA1 peptides may serve as a very early indicator of cognitive impairment in contact sport athletes that precedes manifestation of GluA1 degradation. Another limitation of our study is the relatively small number of participants who sustained a concussion during the season. This limited our ability to examine classification accuracy of GluA1 autoantibodies and peptides compared with traditional methods of diagnosis and to determine the benefit of this approach in relation to traditional diagnostic procedures. This also limited our ability to examine whether GluA1 autoantibody values changed during the natural recovery phase after concussive injury. Although the contact sport versus noncontact sport comparisons yielded significant results and suggests a meaningful relationship between GluA1 autoantibodies and subconcussive head trauma, it is important to recognize that head trauma in the contact sport group was not directly measured in any way beyond individual or team report of concussive injury. Future studies would benefit from methods of directly monitoring head trauma in the contact sport group for additional examination of the relationship to GluA1 autoantibodies, as well as increasing sample sizes to allow for larger numbers of formally diagnosed concussions. Finally, we note that nearly 20% of the sample was lost to follow-up. Nine of the 11 athletes (7 contact and 2 noncontact) who were lost to follow-up were from one university. This was related to the limited availability of athletes in the university sports medicine facilities after the end of the season. Multiple attempts to reach the participants utilizing different methods (phone, email, contact through sports medicine staff) were unsuccessful. Future studies will need to take this factor into account to optimize follow-up.

## Conclusions

In this study, we show an increase in blood based GluA1 autoantibodies in contact sport athletes over the course of the season, relative to noncontact sport athletes, irrespective of whether the athletes experienced a concussion. Amongst contact sport athletes displaying elevated GluA1 antibodies, a serum level of ≥4 ng/mL was associated with significantly impaired cognition. Monitoring of blood levels of GluA1 autoantibodies could represent a clinically meaningful biomarker to identify contact-sports athletes at highest risk of cognitive impairment. Additional research, including large-scale replication, is needed to definitively establish whether monitoring GluA1 autoantibodies will help optimize the management of brain health of contact sports athletes.

## Abbreviations Used

ac-tau: tau acetylation
AMPAR: α-amino-3-hydroxy-5-methyl-4-isoxazolepropionic acid receptor
CV: coefficient variation
GFAP: glial fibrillary acidic protein
ImPACT: Immediate Post-Concussion Assessment and Cognitive Testing
NCAA: National Collegiate Athletic Association
NfL: neurofilament light
PCS-R: Post-Concussive Scale-Revised
TBI: traumatic brain injury
TMB: tetramethyl benzidine
